# The influence of cannabis on sexual functioning and satisfaction

**DOI:** 10.1186/s42238-022-00169-2

**Published:** 2023-01-20

**Authors:** Amanda Moser, Sharon M. Ballard, Jake Jensen, Paige Averett

**Affiliations:** 1 Human Development and Family Science, East Carolina University, Greenville, USA; 2 Social Work, North Carolina State University, Raleigh, USA

**Keywords:** Sex, Cannabis, Sensuality, Weed, Marijuana, Sexual pleasure

## Abstract

**Background:**

The purpose of this study was to examine the perceived influence of cannabis on sexual functioning and satisfaction. This study used Kaplan’s and Masters and Johnson’s sexual response cycle (desire, excitement, orgasm, plateau, resolution) and included satisfaction to complete the sexual response cycle. Given increased attention in the research literature to the potential benefits of cannabis and the lack of research on the sexual benefits of cannabis use, the current study was completed.

**Methods:**

Data were collected using the online survey tool “Qualtrics” from a self-selected, convenience sample of adults over the age of 18 who reported previous cannabis use. The survey, developed by the researchers based on previous literature, included demographic questions followed by a scale to measure sexual functioning and satisfaction in relation to cannabis use (*α* = 0.897).

**Results:**

The final sample was 811 participants ranging in age from 18 to 85 years old (*M* = 32.11). The majority of participants were identified as female (*n *= 536, 64.9%), White/Caucasian (*n* = 640, 78.9%), and college educated (*n *= 650, 80.1%). Almost 25% of the participants were identified as LGBTQIA+ (*n *= 187, 23.1%). Most of the participants reported being in a monogamous sexual relationship (*n *= 598, 73.7%). Data were analyzed using descriptive statistics, *t*-tests, one-way ANOVA, and multiple regression. Age and gender were not found to have significant effects on cannabis use and sexual functioning and satisfaction. Over 70% of participants reported increased desire (*M* = 4.05, SD = 0.962) and orgasm intensity (*M* = 4.05, SD = 0.884). Participants who reported masturbating indicated that cannabis enhanced their pleasure while masturbating (*n* = 620, 62.5%). Participants also stated that cannabis enhanced their sense of taste (*n* = 583, 71.9%) and touch (*n* = 576, 71.0%).

**Discussion:**

The results of this study contrast and establish new evidence within the literature. Demographic results indicate that the people who use cannabis are of a wide range of ages, from a variety of occupations, and have differing cannabis use preferences. The inclusion of LGBTQIA + respondents is a strength of this study. Overall, results indicated that both men and women perceived that cannabis use increased their sexual functioning and satisfaction, particularly increased desire and orgasm intensity.

**Conclusion:**

This study updates the current literature on cannabis and sexuality and provides implications for improving sexual quality. Medical implications of this study include the possible use of cannabis for treating sexual dysfunctions, especially within women.

## Introduction

“*Cannabis sativa* L.,” also known as “cannabis” or “marijuana”, encompasses different varieties based on cannabinoid profiles (Small 2017). Cannabis has been historically used as a multi-functional crop including use as a medicine (Mechoulam et al. 2014; Mikuriya 1969; Russo, 2005), an aphrodisiac (Touw 1981), and as a potential treatment for sexual dysfunctions, such as low sexual desire or sexual pain (Dawley et al. 1979; Lynn et al. 2019). There has been increased attention given to the benefits of cannabis in recent years as it has become legal in many states (Han et al. 2018). Despite its many uses and the increased attention, there is a lack of research on the sexual benefits of using cannabis. Therefore, the purpose of this study is to examine the influences of cannabis on sexual functioning and satisfaction. This paper uses the term “cannabis” in reference to all forms of *Cannabis sativa *L*.*, except within data collection where the term “marijuana” is used as a more recognizable term for all audiences.

Sexual functioning is physiological responses associated with the sexual response cycle that includes desire, excitement, plateau, orgasm, and resolution (Kaplan 1974; Masters and Johnson 1966). Sexual satisfaction encompasses both emotional and physical satisfaction (Basson 2001). Sensuality involves the different sensual effects (touch, taste, smell, sound, and sight) that are associated with sex. While sexual satisfaction has been shown to be influenced by sexual functioning and sensuality (Basson 2001), there is support for sexual satisfaction to be considered as a component of the sexual response cycle (Kontula and Miettinen 2016; Pascoal et al. 2018). The sexual response cycle provides a framework for this study to be organized by each phase (desire, excitement, plateau, orgasm, resolution, satisfaction).

This study compliments gender equality and may have implications for closing the orgasm inequality gap in our society (Mintz 2018). The orgasm inequality gap refers to the fact that orgasms are less consistent for women (Mintz 2018), yet research shows that orgasm is important to sexual satisfaction (Kontula and Miettinen 2016; Pascoal et al. 2018). The current research study emphasizes an individual’s sexual functioning and sexual satisfaction and addresses the need to explore options to help women have more regular orgasms. One possibility for increased orgasm frequency is cannabis (Balon 2017). Using cannabis before sex has possibilities for social change by increasing sexual pleasure within our society as previous research indicates beneficial sexual implications, especially for women (Sun and Eisenberg 2017).

## Background

The literature reviewed will be organized by sexual functioning (specifically using the sexual response cycle as a framework), sexual satisfaction, cannabis, and finally cannabis’ influence on sexual functioning and satisfaction.

### Sexual functioning and satisfaction

Masters and Johnson (1966) established the sexual response cycle that includes four phases: excitement, plateau, orgasm, and resolution. Each phase is identified by physiological responses of the body during sex; however, each phase may not be distinguishable from the next and may differ extensively each time and by each individual. Kaplan’s (1979) Triphasic Concept of sexual response included desire as the first stage of the sexual response cycle and Basson (2001) considered sexual satisfaction to be an important component of the sexual response cycle.

Newer research has expanded the sexual response cycle and adds to the original work of Masters and Johnson and Kaplan. Rather than being linear, the sexual response cycle is circular with overlapping phases that follow a variable order and incorporates mental and emotional components, not just physiological responses (Basson, 2005; Cherkasskaya and Rosario 2018).

Sexual desire, also known as libido, is characterized as a sexual drive or interest in sex that lasts throughout the sexual encounter until orgasm or satisfaction is reached (Kaplan 1979). Cherkasskaya and Rosario (2018) found that sexual desire is on a spectrum that varies between absent or diminished to high desire. Without desire, one may not experience the excitement phase or any following stages of the sexual response cycle because one’s mental state has greater implications than one’s physical desire and arousal (Basson 2008) Toates (2009) created the incentive motivation model that considers the “intertwined progression of desire and arousal” that reinforces the idea that desire and arousal are reciprocally reinforcing.

Excitement is characterized by an increase in sexual tension from an unaroused state and occurs as a result of physical and/or psychological sexual stimulation (Masters et al. 1995). Physiological responses that occur during the excitement phase for both sexes include myotonia (increased neuromuscular tension that occurs throughout the entire body, not just the genital region) and vasocongestion (the swelling of bodily tissues in the genital region due to increased blood flow). Vasocongestion can lead to lubrication in women and an erection in men; however, vaginal lubrication alone is not an accurate measurement of arousal. Women may have genital responses such as lubrication or vasocongestion while not experiencing desire (Chivers and Bailey 2005).

During the plateau phase, sexual arousal is increased while sexual tension levels off prior to reaching the threshold levels required to trigger an orgasm (Masters et al. 1979). During orgasm, there is a release of accumulated sexual tension, and the body induces involuntary rhythmic contractions within the genital region. However, an orgasm is a total body response and is not strictly localized to the pelvic region (Masters et al. 1979).

After orgasm, the body enters the resolution phase and returns to its unaroused state. Yet, if a woman maintains sexual arousal, she is physiologically capable of being multi-orgasmic, meaning having more than one orgasm before returning to her pre-aroused state. Men are typically unable to be multi-orgasmic because of the inevitable phase of the refractory period (i.e., the recovery period required for men to orgasm again after orgasm and ejaculation, which typically gets longer with age).

Sexual satisfaction can be defined as an individual’s subjective evaluation of the positive and negative aspects of one’s sexual relationships (Lawrance and Byers 1995) and may be influenced by many factors such as relationship quality, physical health, and overall well-being (Pascoal et al. 2018). Multiple and consistent orgasms and frequent sex were found to be correlated with higher sexual satisfaction (Kontula 2009; Kontula and Miettinen 2016).

While more than 90% of men report usually experiencing orgasm during sex, less than 50% of women regularly experience orgasm during intercourse and only 6% reported always experiencing an orgasm during sex (Kontula 2009; Koontula and Miettinen 2016). Mintz (2018) in her book *Becoming Cliterate* coined the term “orgasm inequality” to describe the phenomenon of men having routine and consistent orgasms, while women do not. Orgasm consistency is significantly related to sexual satisfaction in women. Women who experience orgasm infrequently or not at all report, on average, lower levels of sexual satisfaction (Kontula, 2009; Kontula and Miettinen 2016). This implies that orgasms during sex are expected for men, but a bonus if accomplished for women (Kontula 2009).

### Sex and cannabis

Cannabis has been identified to have sexually stimulating effects and can intensify sexual experiences (Cohen 1982). The cannabinoid profile in cannabis influences sexual functioning and satisfaction as too much tetrahydrocannabinol (THC) may cause more inhibiting effects (Palamar et al. 2018). Due to its muscle relaxant properties (Small 2017), cannabis use may be inhibitory to men’s sexual functioning, yet, does not impair and may be beneficial for women’s sexual functioning (Sun and Eisenberg 2017). Cannabis may indirectly enhance sexual functioning by decreasing anxiety and increasing relaxation and sensory focus (Klein et al. 2012). It also has been found to be independently associated with increased sexual frequency with daily and weekly users having significantly higher sexual frequency compared to never-users (Sun and Eisenberg 2017).

Historically, and among different cultures, cannabis has been suspected to have an aphrodisiac effect increasing desire and sexual arousal among individuals (Chopra and Jandu 1976; Dawley et al. 1979; Halikas et al. 1982; Mayor’s Committee, 1944). Recent studies support this early research with reports of increased receptivity to and interest in sexual activity after using cannabis with women reporting higher rates of increased desire from cannabis use as compared to men (Androvicova et al. 2017; Lynn et al. 2019). Research has also found that cannabis users intentionally used cannabis for increased sexual desire as well as to decrease pain associated with sex (Green et al. 2003; Lynn et al. 2019).

Cannabis may also have implications during the excitement phase of the sexual response cycle which is characterized by the attainment of an erection in men and vaginal lubrication in women (Masters and Johnson 1966). Using cannabis has been reported to cause the inability to achieve and maintain an erection among men (Chopra and Jandu 1976; Masters et al. 1979) with a higher likelihood of developing erectile dysfunction among habitual users (Aversa et al. 2008). Foreplay could be considered an important part of the excitement stage and Palamar et al. (2018) found that cannabis use can increase the chances and duration of foreplay. Cannabis is also a vasodilator and because there are cannabinoid receptors in the genital region (Small 2017), cannabis may cause vasocongestion (i.e., lubrication) within female users. However, there is contradictory evidence on the influence of cannabis on female lubrication (Masters et al. 1979; Palamar et al. 2018).

During the plateau stage, which occurs after excitement but before orgasm, the vasocongestion response is at its peak in both men and women and the man’s penis is at its full-potential erection (Masters and Johnson 1966). Men are more likely to report increased duration of intercourse when using cannabis compared to women (Palamar et al. 2018; Weller and Halikas 1984). However, time may be *perceived* to last longer when using cannabis due to the altered time effect of cannabis use (Chopra and Jandu 1976; Kaplan, 1974; Palamar et al. 2018) or this may be due to increased time spent during foreplay when couples may engage in sexual exploration and try new behaviors while using cannabis (Palamar et al. 2018).

Orgasm is the release of sexual tension and cannabis use may contribute to more prolonged and pleasurable orgasms (Androvicova et al. 2017; Halikas et al. 1982). However, men’s daily cannabis use has been associated with inability to reach orgasm and reaching orgasm too quickly or too slowly (Smith et al. 2010). Those who are able to orgasm when using cannabis have also reported an increase in the quality and intensity of the orgasm, which was found to be especially apparent for men (Weller and Halikas 1984; Halikas et al. 1982; Palamar et al. 2018).

Cannabis use before sex has been reported to enhance sexual enjoyment and pleasure for individuals, including oral sex (Dawley et al.1979; Halikas et al. 1982; Traub 1977). Sensuality involves the senses (taste, touch, smell, sound, and sight) and, for the purpose of this study, is incorporated as an aspect of sexual satisfaction. Cannabis has continuously been reported to enhance taste and touch but seems to have less of an effect on hearing, smell, and sight (Koff 1974; Masters et al. 1979; Halikas et al. 1982; Weller and Halikas 1984). Increased sensation and sensuality have been found to be related to cannabis use which may be related to length and intensity of intercourse (Palamar et al. 2018). Cannabis use before sex has been associated with more tender, slower, and compassionate sexual acts while also feeling more relaxed with their partner (Palamar et al. 2018).

There is a need for updated research as cannabis use is becoming more prevalent due to legalization (Substance Abuse and Mental Health Services Administration 2018). The majority of existing literature is outdated and some of it is contradictory, such as the physiological effects of cannabis on sexual functioning and satisfaction.

### Research questions

The following exploratory research questions were proposed based on findings from previous literature as well as variables that have not been reported in previous literature: (a) Are there differences between men and women who use cannabis and their perceptions of sexual desire, orgasm intensity, and sexual satisfaction? (b) Does cannabis affect men’s ability to achieve and maintain an erection? (c) Does cannabis use affect women’s orgasm frequency? (d) How does cannabis use affect pleasure while masturbating? (e) What effect does gender, age, duration of cannabis use, intentionality, frequency of cannabis use, and cannabis form have on predicting sexual functioning and satisfaction?

## Methods

This study was approved through the East Carolina University Institutional Review Board and was a self-report survey administered through the online software Qualtrics. Recruitment was purposeful and used snowball sampling. A brief description of the research and the survey were posted on the lead investigator’s personal social media pages (Facebook, Twitter, Instagram, and Tumblr) with encouragement to share with others to increase the sample size. It was also shared on various Facebook groups related to cannabis, cannabidiol (CBD), alternative medicine, and related groups and emailed various cannabis organizations (e.g., medical and legal advocacy organizations) asking members to share the study information on their webpages or through email listservs. The study was voluntary and consent was obtained from all participants. Age and previous cannabis use were the first two questions on the survey to verify inclusion criteria (over 18 years old and have used cannabis in the past). Data collection was open for approximately 5 weeks in January 2019.

### Measures

Study recruitment materials and questions in the survey used the term “marijuana” to refer to all forms of cannabis because it is a widely recognized term. The survey included demographic questions followed by a comprehensive scale developed by the researchers to measure sexual functioning and satisfaction in relation to cannabis use in a manner that used easy to understand format and phrasing.

#### Cannabis use

The questions regarding cannabis measured intentionality of use, benefits of use, where cannabis was obtained, forms used (e.g., flower, wax, etc.), frequency, and duration of use. Sensuality is a construct composed of the five senses. The question measuring cannabis forms asked participants to “check all that apply.’’ To analyze how each form (flower, wax, oil, edible, topical) varied by scale score, each form selected was treated as a separate variable. A dichotomous variable for each of the five forms was created with 1 indicating that form was used by the participant and 0 indicating that it was not used. The frequency of cannabis use question was re-coded to be in the same direction as the other questions with a higher score indicating greater frequency.

#### Sensuality

Previous literature suggests that relaxation enhances sensuality so one item was included to measure relaxation during sex when using cannabis (Palamar et al. 2018). Sensuality was measured with five items with Likert scale response options ranging from *significantly decrease* to *significantly increase*.

#### Masturbation

Masturbation was included to measure sexual functioning and satisfaction with participants who use cannabis for self-pleasure purposes or may not have a sexual partner. Three questions were asked about masturbation: whether or not participants masturbate, if participants use cannabis before masturbating, and if so, how cannabis affects their pleasure while masturbating.

#### Sexual functioning and satisfaction

A scale was developed to measure the participants’ sexual functioning and satisfaction based on the incorporated framework (desire, arousal, orgasm, resolution, satisfaction) to analyze how cannabis influences each stage. This scale was developed as a direct and complete measure to analyze how cannabis specifically influences one’s sexual functioning and satisfaction through each sexual response phase and overall satisfaction in a clear and concise format. The scale consisted of 14 items using the response options ranging from *significantly decrease* to *significantly increase*. These items were influenced by the following empirical studies: Dawley et al. (1974); Koff (1974); and Weller and Halikas (1984). Following development of the scale, all authors reviewed it for accuracy and clarity and to ensure that it adequately reflected current theory and research on sexual response, functioning, and satisfaction.

Arousal was measured with two questions for men (achieving and maintaining an erection) and one question for women (lubrication). In order to have a consistent number of items for both men and women, a new variable was created to measure arousal using one item measuring the ability to achieve an erection for men and one item measuring lubrication for women. The item on maintaining an erection was not used since lubrication and achieving an erection are analogous. The final scale included twelve items (see Table 1) with an internal reliability of 0.897.

**Table 1:** Independent-samples *t*-tests of individual items of the sexual functioning and satisfaction scale

Item	Men *M* (*SD*)	Women *M* (*SD*)	Overall *M* (*SD*)
How does using marijuana affect your *relaxation* during sex?*	4.30 (0.830)	4.45 (0.778)	4.39 (0.801)
How does using marijuana influence your *desire* to have sex (libido, sex drive)?*	3.95 (0.963)	4.10 (0.952)	4.05 (0.962)
How does using marijuana influence your *intimacy/emotional closeness* during sex?	4.06 (0.844)	4.08 (0.930)	4.07 (0.898)
How does using marijuana influence your *physical pleasure*?	4.36 (0.803)	4.31 (0.844)	4.33 (0.830)
How does using marijuana influence your *frequency of sex* (how often you engage in sex)?	3.55 (0.865)	3.54 (0.862)	3.54 (0.860)
How does using marijuana influence your *variety of sexual activities* (i.e. locations, positions, times)?	3.63 (0.813)	3.56 (0.877)	3.58 (0.859)
How does using marijuana influence your *ability to orgasm*?*	3.48 (1.00)	3.86 (0.978)	3.72 (1.00)
How does using marijuana influence your *intensity of orgasm* (how strong the orgasm is)?	4.12 (0.822)	4.01 (0.914)	4.05 (0.884)
How does using marijuana influence your ability to have *more than one orgasm* per sexual encounter (multi-orgasmic)?*	3.45 (0.819)	3.67 (0.901)	3.59 (0.879)
How does using marijuana influence the *duration of sex* (how long sex lasts)?*	3.89 (0.928)	3.59 (0.856)	3.69 (0.894)
How does using marijuana influence your *ability to repeat sex* after orgasm?	3.48 (0.837)	3.43 (0.873)	3.45 (0.858)
Arousal			3.45 (1.01)
Males – How does cannabis influence your ability to *achieve* an erection (boner)?	3.57 (0.892)		
Females – How does using marijuana influence your *vaginal lubrication* (wetness of vagina)?		3.39 (1.05)	

Means range from 1 (significantly decreases) to 5 (significantly increases) with 3 being“does not change”

*p < .05

#### Covariates

Basic demographic information collected included sex/gender, race, LGBTQIA + status, state of residency, education level, relationship status, and socioeconomic status. Participants indicated sex/gender by choosing one of three response options: male, female, or other. Eight response options were provided to measure race: White/Caucasian, Black/African American, Hispanic, Asian, Native American, Pacific Islander, Biracial, and Other. LGBTQ + status was measured by asking participants if they identified as LGBTQ + by choosing yes, no, or prefer not to answer. A drop-down menu was provided for state of residency. Education level was measured in a single item with seven response options ranging from “less than high school diploma or GED’’ to “Ph.D/Doctorate.” Relationship status was measured with a single item with the following four response items: (a) In a monogamous relationship with one person, (b) In an open relationship, (c) Casually hooking up, (d) Not engaging in sexual activity with anybody. Socioeconomic status was measured using the participants’ occupation and annual income which were open-ended questions.

### Analysis plan

Descriptive statistics were used to determine the effect of cannabis use on pleasure during masturbation. Descriptive statistics and independent-samples *t*-tests using individual items from the sexual functioning and sexual satisfaction scale were used to address the first four research questions. Prior to conducting the regression analysis, a Pearson Correlation was performed to examine associations between variables (age, gender, duration of cannabis use, form of cannabis, intentionality of using cannabis prior to sex, and frequency of cannabis use). The results of these preliminary analyses informed the inclusion of variables in the multiple regression. A multiple linear regression was then calculated predicting participants’ scores on the sexual functioning and satisfaction scale based on age, gender, duration of cannabis use, form (flower, wax, oil, edible, topical), and frequency of cannabis use.

A one-way ANOVA was conducted to compare the effect of intentionality on and the sexual functioning and satisfaction scale. Intentionality was measured using one item asking if participants intentionally used cannabis before having sex which had two response options, “yes” or “no”. All statistical analyses were performed using SPSS Statistics V28 (IBM Corporation).

## Results

### Sample description

The original sample size was 1299 participants. Participants (*n* = 133) were removed from the study if they were under the age of 18 or indicated that they had never used cannabis. Another 355 participants did not answer the sexual functioning and satisfaction scale questions resulting in a final sample size of 811 for this study. Analyses were conducted to compare those who had not answered the dependent variable questions and thus excluded from this study (*n* = 355) with those who answered dependent variable questions and were included in the study (*n* = 811). These analyses revealed no significant association between race or ethnicity with inclusion in the study, X2 (7, 1165) = 9.974, *p* = .190, or between sex or gender with inclusion in the study, X2 (2, 1165) = 2.024, *p* = .364. However, a* t*-test revealed that there was a significant difference in age between those included and those who were not included, *t *(1159) = 1.898, *p* = .029. Those included in the study (m = 32.09 years) were older than those excluded (m = 29.27 years) which may have reflected greater comfort in responding to sensitive questions regarding sexual behavior and cannabis use.

Participant ages ranged from 18 to 85 years old (*M* = 32.11). The majority of the participants stated their sex/gender as female (*n* = 536, 64.9%), but the sample also included men (*n* = 277, 34.2%) and those that identified as other (*n* = 8, 1.0%). Most of the participants stated being White/Caucasian (*n* = 640, 78.9%) had at least some college education (*n* = 650, 80.1%) and almost 25% of the participants identified as LGBTQIA+ (*n* = 187, 23.1%). A variety of occupations were represented in this study, including police officers, professors, and stay at home moms. The sample included at least one individual from each state, except South Dakota and Wyoming, and also included individuals from D.C., Puerto Rico, and participants (*n* = 104) that resided outside the USA. Most of the participants reported being in a monogamous sexual relationship (*n* = 598, 73.7%).

### Cannabis use

Over half of the participants reported using cannabis daily (*n* = 509, 62.8%), for recreational and medicinal purposes (*n* = 468, 57.7%), and intentionally using before engaging in sex (*n* = 485, 59.8%). A majority of participants have used cannabis at least a few years (88%; *n* = 714). Almost all participants indicated using cannabis in the form of flower (i.e., pot, weed) (95.9%; *n* = 778). Other forms used by participants included edible (59.2%; *n* = 480), oil (48.0%; *n* = 389), wax (36.5%, *n* = 296), and topical (18.0%; *n* = 146). The majority of participants (78.8%) stated that cannabis does not affect their sexual decision making (*n* = 639) and that cannabis *slightly increases* or *significantly increases* relaxation during sex (87.7%; *n* = 711). Results of the Pearson correlation indicated that there was a strong positive association between age and duration of cannabis use (*r* = .457, *p* = .000), age and frequency of cannabis use (*r* = .167, *p* = .000), and frequency of cannabis use and duration of cannabis use (*r* = .239, *p* = .000).

### Sensuality

Many participants stated that cannabis *slightly increases* or *significantly increases* enhancement of sense of taste (*n* = 583, 71.9%) and 71.0% stated that cannabis *slightly increases* or *significantly increases* their sense of touch (*n* = 576). The majority of participants stated that the enhancement of the following senses does not change with cannabis use: smell (53.3%; *n* = 432), sight (57.2%; *n* = 464), and hearing (56.7%; *n* = 460). Over 70% of participants (*n* = 583) reported that taste was slightly or significantly enhanced when using cannabis (*M* = 3.96, *SD* = 0.943). Similarly, over 70% (*n* = 576) reported that touch was slightly or significantly enhanced when using cannabis (*M* = 4.02, *SD* = 0.906). Table 2 provides mean scores for enhancement of the five senses.

**Table 2:** Mean scores of cannabis use and effect on sensuality by gender

Sense	Men *M* (*SD*)	Women *M* (*SD*)	Overall *M* (*SD*)
Taste	4.02 (0.928)	3.93 (0.949)	3.96 (0.943)
Touch	4.00 (0.905)	4.03 (0.911)	4.02 (0.906)
Smell	3.33 (0.895)	3.28 (0.849)	3.30 (0.865)
Sight*	3.12 (0.817)	2.97 (0.791)	3.02 (0.803)
Hearing*	3.42 (0.889)	3.22 (0.797)	3.29 (0.832)

Means range from 1 (significantly decreases) to 5 (significantly increases) with 3 being “does not change”

*p < .05

### Masturbation

In examining the effects of cannabis use while masturbating, the majority of the participants stated that they masturbate (88.3%; *n* = 716). Of the participants who stated that they masturbate, 76.4% reported using cannabis before masturbating (*n* = 620) and 62.5% indicated that cannabis slightly increases or significantly increases pleasure while masturbating (*n* = 507).

### Sexual functioning and satisfaction

Over 70% of men and women (*n* = 601) reported that cannabis slightly or significantly increases desire (*M* = 4.05, *SD* = 0.962). An independent-samples *t*-test was conducted to compare desire in men and women. The perceived influence of cannabis on sexual desire was significantly higher for women (*M* = 4.10, *SD* = 0.952) as compared to men (*M* = 3.95, *SD* = 0.963); *t*(799) = −2.187, *p* = .029.

Men perceived either no effect or an increased ability to achieve and maintain an erection when using cannabis. Specifically 255 men (93.4%) reported no change or an increased ability to achieve an erection (*M* = 3.57, *SD* = 0.892) and 254 (92.4%) men reported no change or an increase in maintaining an erection (*M* = 3.60, *SD* = 0.928).

Over 70% of men and women (*n* = 582) reported that cannabis slightly or significantly increased orgasm intensity (*M* = 4.05, *SD* = 0.884). An independent-samples *t*-test was conducted to compare cannabis use and orgasm intensity in men and women. There was not a significant difference in the scores comparing men (*M* = 4.12, *SD* = 0.822) and women (*M* = 4.01, *SD* = 0.914); *t* (798) = 1.586, *p* = .113. However there was some support for orgasm frequency among women with over 40% of women (*n* = 356) reporting increased ability to have more than one orgasm per sexual encounter (*M* = 3.67, *SD* = 0.901).

Using descriptive statistics of the scale, men and women reported increased sexual satisfaction (*M* = 3.825, *SD* = 0.613). *T*-test analysis indicated that there was no significant effect based on gender, *t*(801) = − 0.187, *p* = .852. However, because there were significant gender differences in other individual items, gender was included in the regression analyses. A multiple linear regression was calculated predicting participants’ scores on the sexual functioning and satisfaction scale based on age, gender, duration of cannabis use, form (flower, wax, oil, edible, topical), and frequency of cannabis use. The regression equation was significant (*F*(9,789) = 2.582, *p* = .006) with a *R*^2^ of 0.029. The forms wax and flower were significant predictors with topical forms approaching significance (Table 3). A one-way ANOVA was conducted to compare the effect of intentionality of cannabis use prior to sex on the sexual functioning and satisfaction scale. There was a significant effect of intentionality on the scale at the *p* < .05 level [*F*(1,806) = 4.938, *p* = .000] with those intentionally using cannabis before sex having higher scores on the sexual functioning and satisfaction scale.

**Table 3:** Results from linear regression model predicting effects of cannabis use on sexual functioning and satisfaction

Predictor	B	SE	*β*	*t*	*P*
Constant	3.518	0.144		24.503	0.000
Gender	0.021	0.046	0.016	0.451	0.652
Age	0.003	0.002	0.061	1.462	0.144
Duration of cannabis use	− 0.027	0.022	− 0.050	-1.229	0.219
Frequency of cannabis use	− 0.001	0.016	− 0.003	− 0.083	0.934
Form—flower	0.235	0.111	0.077*	2.126	0.034
Form—wax	0.131	0.053	0.103*	2.484	0.013
Form—oil	− 0.013	0.049	− 0.010	− 0.261	0.794
Form—edible	0.050	0.048	0.040	1.039	0.299
Form—topical	0.107	0.061	0.067	1.767	0.078
*R* ^2^		0.029			
*F*		2.582*			

*p < *.05*

## Discussion

This nationwide study had a large sample size with the majority of participants being White college educated women. The inclusion of LGBTQIA + individuals is a strength of this study with almost 25% of the sample identifying as LGBTQIA+. Over half the sample (*n* = 485) reported intentional use of cannabis prior to engaging in sexual activities. Results indicate that the people who use cannabis are of a wide range of ages, from a variety of occupations, and have differing cannabis use preferences. This demographic profile of our sample aligns with previous research that indicates cannabis users vary in age and tend to be non-Hispanic White (Han et al. 2017; Mauro et al. 2017; O’Connell and Bou-Matar 2007). However, our sample differs from recent research regarding sex/gender and relationship status. Although approximately two thirds of our sample were women, Carliner et al. (2017) found that men continue to use at higher rates than women despite the fact that cannabis use has increased for both men and women. Almost 74% of our sample reported being in a monogamous relationship which does not align with recent research that found that regular cannabis users were less likely to be in a relationship (Chan et al. 2021). These differences in our sample as compared to previous research on the sex/gender and relationship status of cannabis users suggest that caution should be used when generalizing results in regard to these demographic characteristics.

### Sexual functioning and satisfaction

An important contribution of this study is the high reliability (*α* = 0.897) for an expanded sexual functioning and satisfaction scale which incorporated Kaplan’s phase of desire, Masters and Johnson’s model (excitement, plateau, orgasm, resolution), and sexual satisfaction as the final stage. This comprehensive scale moves beyond the physiological effects (e.g., achieving an erection) and incorporates overall sexual functioning and satisfaction. The creation of the scale was crucial to gain a comprehensive oversight on aspects of sexual functioning and satisfaction with the ability to analyze and report how cannabis affects various sexual responses. The scale also incorporates the influence of cannabis on sexual functioning and satisfaction, as opposed to a scale that only measures sexual functioning and/or satisfaction.

In contrast to early literature (Koff 1974; Weller and Halikas 1984), no gender differences were found in regard to cannabis use and overall sexual functioning and satisfaction. Results from this study indicated that both men and women see benefits from using cannabis before sexual intercourse or masturbation. However, *t*-tests reveal that there were gender differences with the specific scale items of desire, relaxation during sex, and ability to orgasm. Decreased ability to orgasm could be influenced by both reduced desire and difficulty relaxing during sex. Therefore, if cannabis use allows women to relax and increases desire, they may then have improved orgasm capacity.

Many of the results were consistent with existing literature. One notable exception is men’s ability to achieve and maintain an erection due to cannabis. Previous literature stated that men would have a more difficult time achieving and maintaining an erection when using cannabis, possibly due to the muscle relaxation properties of cannabis (Masters et al. 1979). The current study found that men did not report a decreased ability to achieve and maintain an erection. However, due to the self-report nature of this survey, social desirability may have prevented them from reporting erectile issues.

Similar to existing literature (Androvicova et al. 2017; Lynn et al. 2019), both men and women perceived increased desire and orgasm intensity when using cannabis. Women reported increased ability to have more than one orgasm per sexual encounter, which is similar to previous findings (Weller and Halikas 1984). These results align with the increased relaxation when using cannabis; those who use cannabis report being more relaxed, whether mental or physical, which would improve overall sexual functioning and pleasure. There was no difference in sexual functioning and satisfaction scale scores by age. This indicates that despite age, individuals still report sexual benefits from using cannabis. The age of the sample ranged from 18 to 85, suggesting that cannabis use may have benefits across the lifespan. The positive correlations between age and duration of cannabis use and between age and frequency of cannabis use further support the idea of regular use throughout the lifespan. Additionally, the positive correlation between individuals who have used cannabis for a longer amount of time (duration) and frequency of use means that those who use more cannabis more often were more likely to have been using cannabis for a longer period of time. However, neither duration or frequency of use influenced sexual functioning and satisfaction. People that identify as LGBTQIA + did not differ with cannabis use as one’s sexual functioning and satisfaction is not generally impacted by sexual orientation.

Those who reported intentionally using cannabis before sex had significantly higher scale scores than those who reported not intentionally using cannabis before sex. This can be interpreted as those who intentionally used cannabis before sex perceived a greater benefit to their sexual functioning and satisfaction compared to those who do not intentionally use cannabis before sex. These results may be because of the mental mindset that using cannabis will increase pleasure due to the aphrodisiac notions of cannabis rather than a true physiological effect. However, the relaxation effects of cannabis may contribute to increased desire or reduced inhibitions that might contribute to increased sexual functioning and satisfaction.This also aligns with Palamar et al. (2018) who found that cannabis use can result in more and longer foreplay which can also contribute to positive sexual functioning and seuxual satisfaction. Individuals may also intentionally use cannabis before sex thinking that cannabis use helps with any sexual issues that they have, therefore increasing their sexual functioning and satisfaction.

While dosage could not be measured, forms of cannabis can give an indication of dosage, which has been found to have an impact on sexual functioning (Palamar et al. 2018). Although duration and frequency of cannabis use were not significant predictors, the forms of wax and flower predicted increased sexual functioning and satisfaction. While there is no literature on specific cannabinoid profiles regarding sexual functioning and satisfaction, some products may have a greater influence on the physiological effects and overall satisfaction of sex due to the THC potency and cannabinoid profile.

Sensuality is an important aspect of sexual intercourse as it relates to the five senses. During sex, one uses many, if not all, of their senses. Men and women reported increased enhancement to touch and taste when using cannabis, which is consistent with previous literature (Weller and Halikas 1984). The enhancement of taste and touch could increase overall sexual functioning and satisfaction because these are two senses that are heavily used during sexual intercourse.

### Implications

This study has the potential to impact policy, medicine, and practice by providing support for policy change and legalization advances for cannabis use. Increased access to cannabis may facilitate more research on its effects. Medical implications of this study include the possible use of cannabis for treating sexual dysfunctions, especially with women. Women with vaginismus (i.e., painful intercourse) may benefit from the muscular relaxation and increased sexual functioning that results from cannabis use, while women with decreased desire could also see possible benefits (Lynn et al. 2019).

Finally, regarding practice, results from this study suggest that cannabis can potentially close the orgasm inequality gap (Mintz 2018). The orgasm inequality gap states that men statistically are more likely to orgasm per sexual encounter compared to women (Kontula, 2009). Women may be more likely to orgasm when using cannabis before sexual encounters, which could contribute to equity in the amount of sexual pleasure and satisfaction experienced by both women and men. Sex therapists could incorporate use of cannabis in states where it is currently legal.

### Limitations

While this study had a large sample size and was able to report evidence that has not been found in the literature, there were some limitations. Although the survey was internally reviewed multiple times by all members of the research team, it was not pilot-tested or externally validated. The sample was a convenience sample of individuals who self-selected to participate in the study which may cause selection bias. Additionally, participants were asked to retrospectively self-report based on many years which could result in recall bias. The collection of data by self-report rather than direct observation results in self-report bias in that results are measuring participants’ perceptions of the effects of cannabis rather than the collection of physiological data. Respondents were largely college educated White women, so this study does not represent the majority of US cannabis users.

Dosage was not measured and many individuals are unaware of the amount and potency of cannabis that they are consuming. This is especially true for individuals who do not live in a state where cannabis has been legalized and where all products bought from a regulated dispensary are labeled. Social desirability may be another limitation to this study because of the sensitive nature of the survey questions. Participants may have answered in a desirable manner, particularly related to questions related to erection. This study did not measure medications, mental health status, and other predictors of sexual functioning (Basson 2001; Cherkasskaya and Rosario 2018). Chronic cannabis use has been found to have possible effects (Aversa et al. 2008; Hall, 2014), which this study did not extensively evaluate. Also, several variables were measured using single items and although the scale created had high reliability, it does not have established validity.

### Future research

Cannabis has not been studied extensively, partly because of legalization barriers. This is especially true regarding the intersection of cannabis and sexual functioning and satisfaction. This study found that duration of cannabis use or frequency of cannabis use does not predict sexual functioning. However, previous literature indicates that daily and habitual users reported erectile difficulties in men (Aversa et al. 2008). Future research should focus on men’s frequency and duration of cannabis use in regard to their sexual functioning. Additionally, age was positively correlated with both duration of cannabis use and frequency of cannabis use and the interaction between these three variables should be researched further.

Future cannabis research should focus on specific cannabinoid profiles, methods, and forms to indicate which has greatest sexual impact and implications. Clinical research to study this would be most accurate due to the social desirability effect of self-report surveys. Future research would also benefit from reviewing the endocannabinoid system and its impact on sexual functioning and satisfaction.

## Conclusion

This study extended the limited literature regarding the influence of cannabis on sexual functioning and satisfaction. Results help to update the literature on cannabis and sexuality and contribute to implications for advancing policy, medicine, and practice. Expanding the sexual response cycle to include desire and sexual satisfaction provided a useful framework for this study and results supported this expanded model. Overall, cannabis use tends to have a positive influence on perceived sexual functioning and satisfaction for individuals despite gender or age and cannabis might help to decrease gender disparities in sexual pleasure.

## Data Availability

The datasets used and/or analyzed during the current study are available from the corresponding author on reasonable request.

## Abbreviations

THC: Tetrahydrocannabinol
CBD: Cannabidiol
LGBTQIA+: Lesbian/gay/bisexual/transgender/queer or questioning/other
